# Protocol for a multicenter study on effectiveness and economics of the Back At work After Surgery (BAAS): a clinical pathway for knee arthroplasty

**DOI:** 10.1186/s12891-023-06203-5

**Published:** 2023-03-16

**Authors:** Daniël O. Strijbos, Geert van der Sluis, Wim F. C. van Houtert, A. Carlien Straat, Yvonne van Zaanen, Stephan de Groot, Simon Klomp, Wim P. Krijnen, Carolien M. Kooijman, Igor van den Brand, Michiel F. Reneman, Tim A. E. J. Boymans, P. Paul F. M. Kuijer

**Affiliations:** 1 Department of Public and Occupational Health, Amsterdam UMC, University of Amsterdam, Meibergdreef 9, 1105 AZ Amsterdam, the Netherlands; 2 Amsterdam Public Health, Societal Participation and Health, Quality of Care, Van Der Boechorststraat 8, 1081 BT Amsterdam, the Netherlands; 3 Amsterdam Movement Sciences, Musculoskeletal Health, Sports, Rehabilitation & Development, Van Der Boechorststraat 7-9, 1081 BT Amsterdam, the Netherlands; 4 Department of Health Innovations, Nij Smellinghe Hospital Drachten, Compagnonsplein 1, Drachten, 9202 NN the Netherlands; 5 Hanze University of Applied Sciences Groningen, Zernikeplein 7, 9747 AS Groningen, the Netherlands; 6 Elabo, Landdrostlaan 51, 7327 GM Apeldoorn, the Netherlands; 7 a.S.R. Insurances, Archimedeslaan 10, 3584 BA Utrecht, the Netherlands; 8 Department of Orthopaedics, Elizabeth Tweesteden Hospital, Doctor Deelenlaan 5, 5042 AD Tilburg, The Netherlands; 9 Department of Rehabilitation, University Medical Center Groningen, University of Groningen, Groningen, The Netherlands; 10 Maastricht UMC +, Department of Orthopaedics, P. Debyelaan 25, 6229 HX Maastricht, the Netherlands

**Keywords:** Clinical trial protocol, Health plan implementation, Knee arthroplasty, Occupational health service, Orthopedics, Physical modalities, Return to work

## Abstract

**Background:**

Optimizing return to work (RTW) after knee arthroplasty (KA) is becoming increasingly important due to a growing incidence of KA and poor RTW outcomes after KA. We developed the Back At work After Surgery (BAAS) clinical pathway for optimized RTW after KA. Since the effectiveness and cost analysis of the BAAS clinical pathway are still unknown, analysis on effectiveness and costs of BAAS is imperative.

**Method:**

This protocol paper has been written in line with the standards of Standard Protocol Items: Recommendations for Interventional Trails. To assess the effectiveness and cost-effectiveness for RTW, we will perform a multicenter prospective cohort study with patients who decided to receive a total KA (TKA) or an unicompartmental KA (UKA). To evaluate the effectiveness of BAAS regarding RTW, a comparison to usual care will be made using individual patient data on RTW from prospectively performed cohort studies in the Netherlands.

**Discussion:**

One of the strengths of this study is that the feasibility for the BAAS clinical pathway was tested at first hand. Also, we will use validated questionnaires and functional tests to assess the patient’s recovery using robust outcomes. Moreover, the intervention was performed in two hospitals serving the targeted patient group and to reduce selection bias and improve generalizability. The limitations of this study protocol are that the lead author has an active role as a medical case manager (MCM) in one of the hospitals. Additionally, we will use the data from other prospective Dutch cohort studies to compare our findings regarding RTW to usual care. Since we will not perform an RCT, we will use propensity analysis to reduce the bias due to possible differences between these cohorts.

**Trail Registration:**

This study was retrospectively registered at clinicaltrails.gov (https://clinicaltrials.gov/ct2/show/NCT05690347, date of first registration: 19–01-2023).

**Supplementary Information:**

The online version contains supplementary material available at 10.1186/s12891-023-06203-5.

## Background

Optimizing return to work (RTW) after knee arthroplasty (KA) is becoming increasingly important due to the growing incidence of KA and poor RTW outcomes after arthroplasty. The incidence of KA is predicted to rise substantially in the coming decades in countries with established market economies [1–4]. The largest increase is expected among patients of working age [5]. For instance, in 2030 the United States of America will be the first country where the majority of patients getting KA are of working age, followed by Great Britain in 2035 [3, 4]. Although outcomes regarding pain relief and improved knee function after KA are satisfactory, RTW among patients getting KA is relatively low. Prospectively reported non-RTW rates are 33% within six months after KA and 13% within 12 months in the Netherlands [6]. Also, the economic burden of knee osteoarthritis in the Netherlands is high. The average annual cost for the Dutch workforce due to sick leave for knee osteoarthritis was 26.9 million euros between 2015 and 2017 [7]. Considering the increase in the demand from patients seeking KA, the low RTW outcomes after KA, and a high economic burden, care optimization for patients getting KA with a focus on RTW is essential.

A closer collaboration between professionals in medical care (e.g. orthopedic surgeon, physical therapist) and occupational care (e.g. occupational physician) for working-age patients with KA might improve their RTW probabilities after surgery [8]. From the patient perspective, an individual tailored, integrated (health care and work-directed), comprehensive (from pre-operative to RTW) clinical pathway should preferably include integrated and coordinated care from four domains: (i) rehabilitation (e.g. physical therapy); (ii) patient support (e.g. setting proper goals); (iii) occupational support (e.g. proper build-up modified work); and (iv) medical support (e.g. more personal guidance from the hospital) [9, 10]. Unfortunately, proven effective work-directed care is currently not available for patients receiving KA [11, 12].

Recently, an integrated clinical pathway called Back At work After Surgery (BAAS) was started, aiming to improve RTW after KA. BAAS was proven feasible in 2021 in practice (Additional file 1: Appendix I) [13]. The development of this care pathway was based on two pillars [14]. The first pillar was a timely combination of medical and occupational care, and the second pillar was enhancing patient participation during the clinical pathway. In this way, interdisciplinary communication was improved by making RTW an explicit outcome of the rehabilitation, which might accelerate both rehabilitation and RTW. The next step is to analyze the effectiveness and costs of the BAAS clinical pathway to RTW.

To the best of our knowledge, this is the first study on work-directed and patient-centered care after KA systematically involving health care experts other than an orthopedic surgeon and a physical therapist. Timely involvement of occupational care experts seems to be an important evidence-based prerequisite to ensure that the BAAS clinical pathway can be more effective on RTW after KA than usual care [6, 13, 15]. However, since the true effectiveness and costs or benefits of the BAAS clinical pathway are still unknown, analysis on effectiveness and economic evaluation of BAAS is expedient. Our hypotheses are that an integrated clinical pathway with a focus on RTW after KA is more effective on RTW than usual care, and has a positive economic evaluation.

This multicenter study has two aims. Firstly, to assess the effectiveness of the BAAS clinical pathway for RTW compared to usual care. Secondly, to evaluate the economics of the BAAS clinical pathway compared to usual care. This paper describes the study protocol for the multicenter study.

## Methods

### Study design

This protocol paper has been written in line with the standards of Standard Protocol Items: Recommendations for Interventional Trails (SPIRIT) [16]. We will perform a multicenter prospective cohort study with patients who have decided to be given a total KA (TKA) or a unicompartmental KA (UKA). To establish generalizability, the BAAS pathway will be performed in two high-volume KA hospitals in the Netherlands serving a representative population of working patients receiving KA. For the effectiveness of BAAS regarding RTW, the results of the study will be matched in pairs and compared to individual patient data on RTW by matched pairs from prospectively performed cohort studies in the Netherlands on care as usual, namely the Expect to Work cohort and the ACTIVE trial [17, 18]. For the economic evaluation, a comparison will be made with individual patient-level data using the care-as-usual group of the ACTIVE trial in the Netherlands [17]. The study has been approved by the Medical Ethical Committee of the Amsterdam UMC, location AMC (reference ID: W21_454 # 21.504) and the Medical Ethical Committees of the local hospitals Nij Smellinghe (NS, reference ID: 19,888) and Elizabeth Tweesteden Ziekenhuis (ETZ, reference ID: L1429.2021).

### Study setting

This study will take place in two hospitals in the Netherlands. The first is Nij Smellinghe (NS) hospital located in Drachten, in the northern part of the Netherlands. NS has approximately 320 clinical beds and three orthopedic surgeons who perform approximately 350 KAs a year. The second is the Elizabeth Tweesteden Ziekenhuis (ETZ), a larger regional hospital located in Tilburg, in the southern part of the Netherlands. ETZ has 940 beds and 6 orthopedic surgeons who perform approximately 700 KAs a year.

### Participants

#### Sample size

A minimal required sample size was calculated using R (version 3.6.3) with a power of 0.8 and a significance level of 0.05. The effect size was calculated with a mean difference of two weeks in comparison to a Dutch study on RTW after KA [15]. With a calculated minimal required sample size of 125 and an expected loss to follow-up of 20%, we intend to include 150 patients (75 in NS and 75 in ETZ).

#### Recruitment

Inclusion criteria for patients are: (ii) being scheduled for primary or revision UKA or TKA by an orthopedic surgeon between January 2022 and July 2023; (ii) having paid work for at least eight hours a week before surgery; (iii) being between 18 and 65 years of age and (iv) having the intention to fully RTW after surgery. Criteria to exclude patients are: (i) receiving more than one medical event within one year that affects work ability after KA; (ii) having a KA for any other reason than knee osteoarthritis; and (iii) having major disabling mental disorders. Patients who do not speak or read Dutch are given the opportunity to fill in the questionnaires with the aid of a translator and to have an interpreter present during the consultations. Patients who are eligible to participate are informed about the study by telephone by the medical case manager (MCM; physical therapist working in the hospital). During an intake consultation with an MCM, the patient information letter, informed consent, and an infographic of the BAAS clinical pathway (Additional file 1: Appendix I) are handed out to the patient. Patients’ questions regarding participation are answered. Patients are given one week to decide whether or not they wish to participate. Patients who opted for the possibility to participate receive a telephone call after one week so that they can ask any additional questions about the study and they will be asked if they want to participate. Participants willing to participate sign the informed consent.

#### Patient timeline

**Fig. 1:**
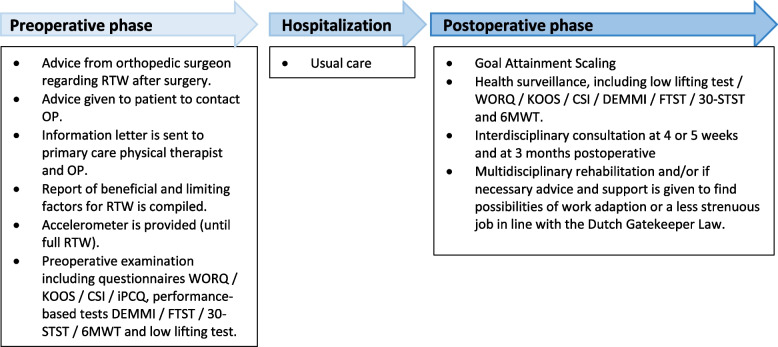
The BAAS clinical pathway. Abbreviations: 6MWT = 6-min walk test, CRT = Chair Rise Time, CSI: Central Sensitization Inventory, DEMMI = de Morton Mobility Index, FTST: Five Time Sit to Stand, iPCQ = iMTA Productivity Cost Questionnaire, KOOS = Knee Osteoarthritis Outcome Score, OP = occupational physician, RTW = RTW, WORQ = Work, Osteoarthritis and Joint-Replacement Questionnaire

Since the analysis on effectiveness and economic evaluation will be conducted on the same cohort, the participant timeline will be identical (Fig. 1 and Table 1).

**Table 1:**
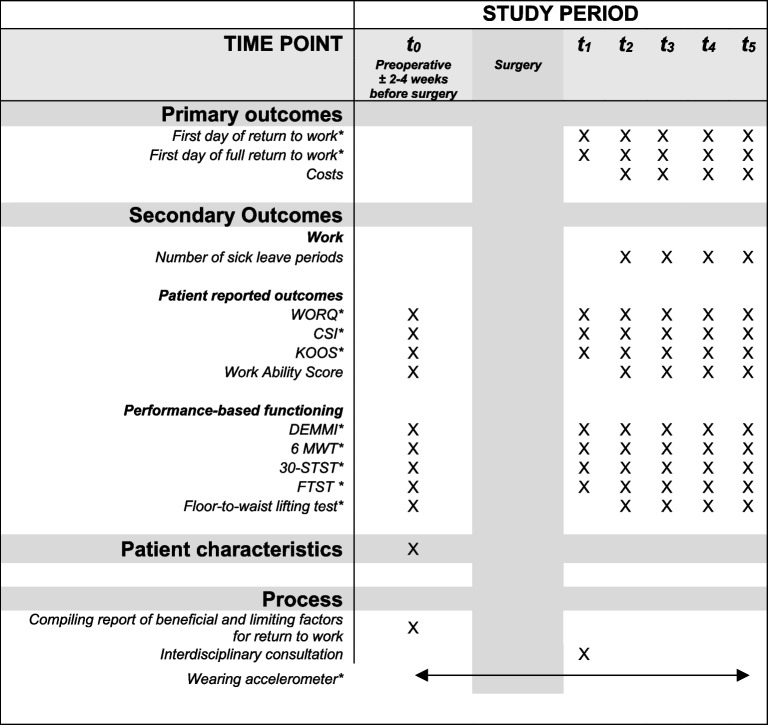
Measurements on different time points

T_0_ = baseline; T_1_ = 6 weeks after surgery; T_2_ = 3 months after surgery; T_3_ = 6 months after surgery; T_4_ = 9 months after surgery; T_5_ = 12 months after surgery

*Abbreviation*: *CSI* Central sensitization inventory, *DEMMI* De Morton mobility index, *FTST* Five time sit to stand test, *iPCQ*, *KOOS* Knee osteoarthritis outcome score, *WORQ* Work Osteoarthritis and joint-replacement questionnaire, *6 MWT* 6-Minute walk test, *30-STST* 30 Seconds sit to stand test. * = follow-up measurements stop after full return to work

### Measures

#### Primary outcomes

For the study on effectiveness, the primary outcomes are first day of RTW and/or first day of full RTW. First day of RTW is defined as: time in days from surgery to the first day of returning to work, regardless of the number of working hours or tasks performed. Full RTW is defined as: time in days from surgery to the first day a patient works the number of hours stated in his or her employment contract regardless of the tasks performed. For self-employed patients, full RTW is defined as: the first day of the week a patient works the number of hours equal to the number of hours he or she worked before surgery. For the first days to RTW the prospective Expect to Work study by Van Zaanen et al. will be used as usual care cohort, and for full RTW the ACTIVE trial comparison group by Straat et al. will be used [17, 18]. Test–retest reliability, minimal clinical importance difference, and minimal detectable difference of self-reported RTW are not described in the medical literature, if we are not mistaken. The effectiveness study of BAAS will be reported according to the Strengthening the Reporting of Observational Studies in Epidemiology (STROBE) guidelines [19].

For the economic evaluation, the primary outcomes are the total costs of the intervention, and the societal and health care cost obtained by a cost-questionnaire at 3, 6, 9 and 12 months according to the guidelines of the Dutch Health Care Institute (Zorginstituut Nederland), in line with the study performed by Straat et al. [17, 20]. The economic evaluation will be reported according to the Consolidated Health Economic Reporting Standards 2022 (CHEERS 2022) and we will use the human-capital method [21].

Costs of the BAAS care pathway intervention include: (i) cost of case managers; (ii) cost of compiling the report by the OCM; (iii) cost of professionals present at the digital interdisciplinary consultation and (iv) cost of the use of the accelerometer program. These costs will be estimated using the micro-costing approach. In other words, the cost estimation will be based on actual resources depleted, which will be assessed using prospective data collection, and will be valued in accordance with the Dutch guidelines for conducting economic evaluations in health care [20]. Societal cost includes occupational health care services, informal care, unpaid productivity loss, absenteeism, presenteeism and other health care services. Absenteeism (e.g. total number of sick leave days, measured prospectively by the MCM) and presenteeism (e.g. lower productivity as compared to normal while at work) will be valued using sex-specific price weights [20]. Unpaid productivity losses (e.g. voluntary work) and informal care are measured by asking the number of hours per week patients were unable to perform unpaid activities in the cost-questionnaire. Unpaid productivity losses and informal care will be valued using a recommended Dutch shadow price [20]. Using consumer price indices, all costs will be converted to the same reference year by using the human-capital method.

For health care costs, all costs according to the formal Dutch health care sector will be obtained, including cost of primary care (e.g. family physician or primary care physical therapist), secondary care (e.g. hospital stays and visits), tertiary care (vocational rehabilitation) and medication. If available, these will be valued using Dutch standard costs [20]. If unavailable, prices of professional health care organizations will be used. Medication use will be valued using prices derived from http://www.medicijnkosten.nl. All costs will be summed and divided by the number of participants, separate for both groups. This will lead to average (95% CI) total costs per participant.

#### Secondary measures

The Work, Osteoarthritis and Joint-Replacement Questionnaire (WORQ) is a questionnaire containing thirteen 0–4-scale questions regarding perceived difficulty with performing work-related knee straining activities, calculated to a 0–100 total score, in which a higher score represents less difficulties performing the activity [22]. The WORQ is a reliable, valid, and responsive questionnaire that can be used to evaluate the impact of knee complaints following KA on patients' ability to work with an inter rater correlation (ICC) of 0.97 [22].

The Knee Injury and Osteoarthritis Outcome Score (KOOS) is a questionnaire containing thirty-seven 0–4 ordered category questions regarding KA-related symptoms, pain, activities, sport participation, and quality of life domains, normalized into a 0–100 total score on every domain, in which a higher score represents less restriction on the given domain [23, 24]. The KOOS demonstrates adequate content validity, internal consistency, test–retest reliability, and construct validity with an ICC of 0.85—0.90 [25].

The Central Sensitization Inventory (CSI) is a questionnaire containing twenty-five 0–4-scale questions regarding pain sensitization-related symptoms in which a higher score represents a higher change on pain sensitization-related symptoms [26]. The Dutch translation of the CSI has four distinguishable domains, has good internal consistency for the total score and three out of four domains, good discriminative power, and excellent test–retest reliability [26].

The Work Ability Score (WAS) consists of the worker’s self-reported current work ability compared to the lifetime best. The score ranges from 0 to 10 and a lower score represents a lower ability to work [27]. The WAS is more user-friendly than the Work Ability Index and has a good and comparable reliability [28].

The iMTA Productivity Cost Questionnaire (iPCQ) is a questionnaire containing cost-related questions regarding health and work [29, 30]. Test–retest reliability, minimal clinical importance difference, and minimal detectable difference of the iPCQ are unknown.

The de Morton Mobility Index (DEMMI) is a 15-item unidimensional mobility instrument that measures static and dynamic balance. The 15 items are recalculated to a 0–100 points score, in which a higher score represents better mobility [31]. The DEMMI has a good validity, a Minimal Detectable Change (90; MDC), and minimal clinical important difference (MCID) of respectively 9 and 10 points [31].

The 6MWT is a walk test in which the maximum walking distance is measured during a period of six minutes [32]. The 6MWT has an MCID of 74.3 m [33].

The 30-STST is an instrument to assess the functional status of patients. A patient is asked to perform as many sit and stand repetitions within 30 s without using their hands. The 30-STST is a valid and reliable instrument and has an MCID of 1.13 [34].

The FTST is a similar test to the 30-STST and measures the time it takes for a patient to perform 5 sit and stand repetitions without using their hands. Because of their similarity, the 30-STST and FTST can be performed simultaneously. The FTST is a valid and reliable instrument and has an MCID of 2.3 s [35].

The floor-to-waist lifting test is a reliable and valid performance-based test to assess a patient’s functional capacity [36, 37]. Reference values to assess whether a patient is restricted regarding functional capacity to RTW are between 16 and 24, dependent on the physical nature of the job [38].

#### Demographics

We will collect age, sex, length, weight, type of surgery (UKA/TKA), side of surgery (left/right), length of stay, working hours per week, working days per week, physical nature of the job, preoperative sick-leave days, breadwinner, being self-employed, comorbidities, and complications after surgery.

### Study intervention

The following BAAS work-directed care is offered to the patient, which was proven feasible (Additional file 1: Appendix I) [13]. The orthopedic surgeon provides information before surgery about returning to work after surgery in terms of expected time to RTW and the known prognostic factors like female gender, affirmative patient-reported work-relatedness of knee symptoms, high physical work demands, high Body Mass Index (BMI), and prolonged preoperative sick absence from work [39, 40]. Next, the orthopedic surgeon refers the patient to the MCM for a preoperative examination. During this preoperative examination, the patient fills out several questionnaires (WORQ, KOOS, CSI, WAS and iPCQ; Table 1) so the MCM gains insight in the preoperative status of the patient (Fig. 1). Then, the patient is subjected to performance-based physical tests to evaluate the functional capacity, namely a floor-to-waist lifting test and functionality tests, including the DEMMI, FTST, 30-STST, and 6MWT. During this preoperative examination, the patient is given information about the perioperative care and has the opportunity to ask questions about the recovery trajectory. The findings of the preoperative examination are used as baseline measurements for setting goals in the postoperative rehabilitation and as reference values for postoperative recovery. Next, the patient receives an accelerometer (PAM 2.0), including the option of feedback from the software application Atris (Peercode B.V.) during the preoperative examination. The movement data is accessible for the patient, the primary care physical therapist, and the MCM. A week later, the MCM calls the patient to discuss the current physical activity assessed by the accelerometer and give advice on the preferred physical preoperative preparation. For example, patients are advised to train functional movements required after surgery, such as walking with a walking aid, or are advised to maintain or increase the current physical fitness level by adhering to the WHO’s latest physical activity guideline [41]. The MCM then contacts the occupational physician to inform him or her about the participation of the patient in this clinical pathway. The patient is advised to consult the occupational physician before surgery. Then, the patient is referred to the occupational case manager (OCM; occupational assessor) by the MCM, to compile a report of beneficial and limiting factors regarding RTW after KA.

During the hospitalization, the patient receives perioperative care as usual according to the KA fast track principles in both hospitals [42]. At NS hospital, patients who have decided to have KA receive an Oxford uncemented Partial Knee (Zimmer Biomet), NexGen LPS-Flex or CR Total KA (Zimmer Biomet) or Medial Rotation Knee total knee replacement (BdH Medical BV) or SAIPH Knee System (BdH Medical BV). At ETZ hospital, patients who have decided to have KA receive a SIGMA Total Knee System (Johnson and Johnson) or an Oxford Partial Knee (Zimmer Biomet).

In both hospitals, patients will receive physical therapy according to usual care while being hospitalized for two or three times a day, with the goal of gaining independence in activities of daily living such as walking, transfers in and out of bed or chair, and walking up and down stairs if necessary [43]. Also, the orthopedic surgeon and physical therapist motivate the patient to train range of motion of the knee to at least full extension (0 degrees) and 90 degrees of flexion. After hospitalization, the patient receives physical therapy from a primary physical therapy setting according to the patient’s preferences and taking into account the Royal Dutch Society for Physiotherapy (KNGF) guidelines for knee osteoarthritis [44]. The physical therapist is informed that the patient is participating in BAAS care pathway, and informed about the content of this pathway through an information letter. A work-related therapy goal is set up from the start of the therapy and monitored every six weeks throughout the whole clinical care pathway using Goal Attainment Scaling (GAS, Fig. 1) by the MCM. Progress in recovery is evaluated through questionnaires (WORQ, KOOS, and CSI), functional capacity test (floor-to-waist lifting test), functional tests (DEMMI, FTST, and 6MWT) and the physical activity level measured by the accelerometer (Table 1). The questionnaires and functional capacity evaluations tests are repeated at 6 weeks and every 3 months after surgery (Fig. 1), with the exception of the floor-to-waist lifting test at six weeks after surgery due to patient safety reasons. Data from the accelerometer is evaluated by the MCM on a weekly basis. Stop criteria for the BAAS clinical pathway are full RTW or, if RTW was not achieved after one year.

An interdisciplinary consultation is held the fourth or fifth week after surgery. The patient, the primary care physical therapist, the MCM, OCM, and occupational physician are invited to participate (Fig. 1). During this consultation, the progress, the attainment of the GAS goal, and RTW plan according to the Dutch Gatekeeper Improvement Act are discussed. If the patient has a job which he or she probably cannot return to, for example because of high knee demands due to prolonged kneeling and squatting, the possibility of work adaptions or even the topic of finding a less physically strenuous job are discussed (Fig. 1). The interdisciplinary consultation is continued if required, for instance based on unfavorable recovery data. If, after three months a delayed recovery is seen based on the patient’s experience and expert opinions of the MCM, OCM, and occupational physician, and supported by questionnaires, functional capacity evaluations and accelerometer data, the patient is referred to a multidisciplinary rehabilitation assessment (Fig. 1). Here, the patient is examined by a rehabilitation physician, occupational medicine specialist, physical therapist, and psychologist to assess barriers for delayed recovery and RTW, and whether the patient is eligible for an interdisciplinary vocational rehabilitation program. If so, the patient receives this interdisciplinary rehabilitation program [45].

The MCM from NS hospital, who is experienced in fulfilling the task of MCM through the previously performed feasibility study, will train two physical therapists working in ETZ. These physical therapists perform the tasks of an MCM in their hospital. Also, the MCMs of ETZ will be trained to perform all functional tests. Every two weeks a digital consultation will be held between all MCMs to discuss progress of the study and any questions that have arisen during the entire study period.

### Data collection

For the effectiveness study, both date of surgery and RTW data are collected by the MCM. In addition to the RTW data, the preoperative data and health surveillance data (Fig. 1) are collected in the same file by the same MCM.

### Data management

For this study, data will be coded and collected in a password-protected Microsoft Excel file. A separate Excel file will refer to the coded data of individual patients that is only accessible by the MCM.

### Statistical methods

#### Effectiveness

The effect of the BAAS care pathway on time to RTW will be statistically tested using survival analysis and gamma regression analysis including bootstrapping using R (version 4.1.0). Missing data will be handled by multivariate imputation using the aforementioned prognostic factors if possible [46]. For the repeated measurements on the time to RTW and/or full RTW, a mixed model will be used to test and estimate the size of the BAAS effect controlling for the following potential prognostic factors of delayed RTW, namely UKA vs TKA, primary vs revision KA, gender, BMI, physical nature of the job, preoperative sick leave, and patient-reported work-relatedness of knee symptoms. Hazard ratios including 95% confidence intervals will be calculated for the intervention group and tested against both control groups. In the absence of an empirically derived MCID, a difference of two weeks will be considered clinically relevant for first day of RTW and one week for full RTW. For interpreting the magnitude of the standard mean difference (SMD), three groups are defined: (i) small (SMD = 0 – 0.2); (ii) medium (SMD = 0.2 – 0.5) and (iii) large (SMD = 0.5–1.0) [47]. Sensitivity analyses on RTW will be performed for the two hospitals. Analyses will be conducted using both univariate and multivariate analyses to assess the effect size of the BAAS care pathway with and without controlling for variables. Lastly, because this control group is not from the same cohort as the intervention group, we will use propensity analysis (matched pairs) to correct for other confounding factors [48]. Potential prognostic factors for delayed RTW like UKA vs TKA gender, BMI, physical nature of the job, preoperative sick leave and patient-reported work-relatedness of knee symptoms will be used for patient matching. Secondary outcomes will be plotted in time and will be tested on differences between the usual care cohorts and the intervention cohort.

#### Economic evaluation

For the economic evaluation, missing data will be imputed using multivariate imputation using the aforementioned prognostic factors if possible [46]. An Ordinary Least Squares regression model with bootstrapping will be used to investigate the differences between the BAAS cohort and the usual care cohort of the ACTIVE trial. Lastly, because this control group is not from the same cohort as the intervention group, we will use propensity analysis to correct for the two different cohorts (intervention and control) [48]. Potential prognostic factors for delayed RTW like UKA vs TKA gender, BMI, physical nature of the job, preoperative sick leave, and patient-reported work-relatedness of knee symptoms will be used for patient matching. Results will be plotted for interpretation. The difference in costs and benefits are clinically relevant when the intervention has more financial benefit in comparison with usual care.

## Discussion

This study describes the protocol to evaluate the effectiveness of the BAAS care pathway for RTW as well as its costs and benefits. To the best of our knowledge, this is the first work-directed and patient-centered care after KA systematically involving health care experts other than an orthopedic surgeon and a physical therapist.

To the best of our knowledge, there is no previous study concerning minimal clinical differences in return to work after surgery. Therefore, we held a consensus meeting with authors DS, GS, TB, PK and MR in which we concluded that a minimal clinical difference of two weeks on start to return to work and one week for full return to work is relevant.

Until now, no core outcome set exists for work participation including RTW to be used in intervention studies [49, 50]. Previous studies on RTW among patients with knee arthroplasty defined RTW in different ways. For example, Hylkema et al. measured the proportion of full RTW within 3, 6 or 12 months by asking the patient via a questionnaire whether they partially or fully returned to work, or not at all [6]. Tilbury et al. measured RTW by asking patients through a follow-up questionnaire what the duration from operation until the first day of RTW was [15]. Systematic reviews on RTW among patients with knee arthroplasty concluded that pooling data on RTW results in heterogeneity due to these varying RTW outcomes. To obtain a valid comparison, we used the same RTW definitions as those used in the prospective studies of Straat et al. and Zaanen et al. [17, 18]. While this RTW definition is needed to compare the results with the prospective studies, it also means that comparisons with other studies (vice versa) should be performed carefully.

The strengths of this protocol are that the BAAS clinical pathway was already proven feasible [13]. This way, not only did we learn that BAAS was feasible to implement, but we also learned important lessons to optimize the BAAS clinical pathway before evaluating its effectiveness, costs, and benefits. Also, the feasibility showed us promising results on the first day of RTW and full RTW in comparison to other Dutch prospective studies on RTW after KA. For example, patients started on average after 6.4 [6–8.1] weeks, in comparison to 12.9 weeks in the study of Tilbury et al. [15]. Patients fully returned to work on average after 12.4 [9.4–14.4] weeks, in comparison to the study of Hylkema et al., where 49% of the patients took more than six months to full RTW [6]. Of course, the present study should show whether these RTW data are reliable and valid. Also, we will use reliable and valid questionnaires and functional tests to assess the patient’s recovery. In addition, patients who experience widespread sensitization after KA are identified early and can be referred for multidisciplinary vocational rehabilitation [45, 51]. Also, by setting personal goals regarding work-related knee-straining activities using Goal Attainment Scaling, all the involved professionals have a focus towards work participation [52]. This probably supports both patient and professional in a timely and better collaboration between professionals within the medical and occupational trajectory. By focusing on these work-related activity goals, we aim to create more awareness in the multidisciplinary team about the values and needs of patients regarding their disability and what he or she is intended to achieve in work.

In transition processes, in which care as usual is replaced by a new care pathway, the essential role of the patients and health care professionals is often overlooked. Therefore, in our study the professionals involved in the care transitions in the two hospitals will collect and scrutinize the data together and, based on the results, determine what kind of action should be performed to enhance RTW in close contact with the patient. One of the consequences of this iterative cycle will be that all professionals as well as the patients involved become partners in achieving an optimal RTW. This way, the embedded scientist who, in daily practice, collects, creates, and reviews knowledge, and investigates the effectiveness of this newly implemented care pathway is an essential actor in creating and implementing future RTW pathways for other groups of working age patients.

Lastly, by using the data from two Dutch cohorts on RTW with similar inclusion criteria, our study is designed to be more cost-effective than a randomized controlled trial.

The limitations of this study protocol are that the lead author has an active role as an MCM in this study. This is why we included patients from a second hospital (ETZ). Also, we will not perform an RCT. We will use the data from two other prospective cohort studies with similar inclusion criteria to compare our findings to usual care. The care for knee arthroplasty is, fortunately, largely standardized in the Netherlands [44, 53]. Also, we will use propensity analysis to reduce the bias due to possible differences between our cohorts and the two comparison cohorts [17, 18].

## Supplementary Information

**Additional file 1**: **Appendix I.** Comic strip of BAAS clinical pathway.

## Data Availability

The datasets used and/or analyzed during the current study are available from the corresponding author on reasonable request.

## Abbreviations

BAAS: Back At work After Surgery
BMI: Body Mass Index
CHEERS 2022: Consolidated Health Economic Evaluation Reporting Standards 2022
CI: Coincidence interval
CSI: Central Sensitization Inventory
DEMMI: De Morton Mobility Index
ETZ: Elizabeth Tweesteden hospital Tilburg
FTST: Five Time Sit to Stand Test
GAS: Goal Attainment Scaling
ICC: Inter rater correlation
IPCQ: IMTA Productivity Cost Questionnaire
KA: Knee arthroplasty
KNGF: Royal Dutch Society for Physiotherapy
KOOS: Knee Injury and Osteoarthritis Outcome Score
MCID: Minimal clinical important difference
MCM: Medical Case Manager
MDC: Minimal detectable change
NS: Nij Smellinghe hospital Drachten
OCM: Occupational Case Manager
RTW: Return to work
SPIRIT: Standard Protocol Items: Recommendations for Interventional Trails
STROBE: Strengthening the Reporting of Observational Studies in Epidemiology
TKA: Total Knee Arthroplasty
UKA: Unicompartmental Knee Arthroplasty
WAI: Work Ability Index
WAS: Work Ability Score
WORQ: Work, Osteoarthritis and Joint-Replacement Questionnaire
30-STST: 30 Second Sit to Stand Test
6 MWT: 6-Minute walk test
