# Supplementary figures and images for: Protocol for a multicenter study on effectiveness and economics of the Back At work After Surgery (BAAS): a clinical pathway for knee arthroplasty
Source: BMC Musculoskelet Disord. 2023 Mar 16;24:199. doi: 10.1186/s12891-023-06203-5 (PMC10018987; doi:10.1186/s12891-023-06203-5)

**Appendix I – Comic strip of BAAS clinical pathway**


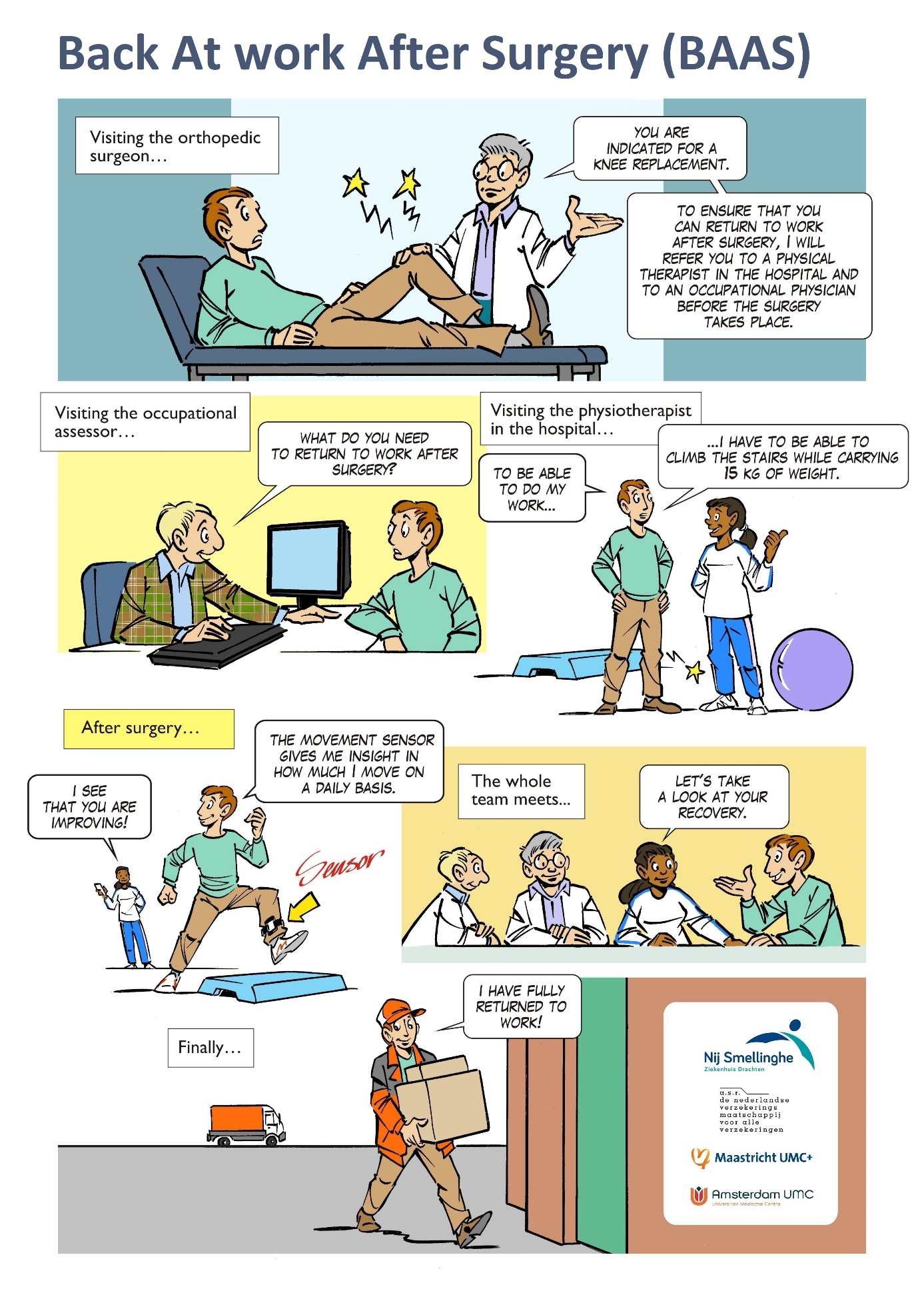

Supplement: Supplementary file 1 — Additional file 1: Appendix I. Comic strip of BAAS clinical pathway. [file 12891_2023_6203_MOESM1_ESM.docx]
